# Diagnostic Delay and Its Predictors in Cluster Headache

**DOI:** 10.3389/fneur.2022.827734

**Published:** 2022-02-10

**Authors:** Byung-Su Kim, Pil-Wook Chung, Byung-Kun Kim, Mi Ji Lee, Min Kyung Chu, Jin-Young Ahn, Dae Woong Bae, Tae-Jin Song, Jong-Hee Sohn, Kyungmi Oh, Daeyoung Kim, Jae-Moon Kim, Jeong Wook Park, Jae Myun Chung, Heui-Soo Moon, Soohyun Cho, Jong-Geun Seo, Soo-Kyoung Kim, Yun-Ju Choi, Kwang-Yeol Park, Chin-Sang Chung, Soo-Jin Cho

**Affiliations:** ^1^Department of Neurology, Daejin Medical Center, Bundang Jesaeng General Hospital, Seongnam, South Korea; ^2^Department of Neurology, Kangbuk Samsung Hospital, Sungkyunkwan University School of Medicine, Seoul, South Korea; ^3^Department of Neurology, Eulji Hospital, Eulji University, Seoul, South Korea; ^4^Department of Neurology, Neuroscience Center, Samsung Medical Center, Sungkyunkwan University School of Medicine, Seoul, South Korea; ^5^Department of Neurology, Severance Hospital, Yonsei University College of Medicine, Seoul, South Korea; ^6^Department of Neurology, Seoul Medical Center, Seoul, South Korea; ^7^Department of Neurology, College of Medicine, The Catholic University of Korea, Suwon, South Korea; ^8^Department of Neurology, Seoul Hospital, Ewha Womans University, College of Medicine, Seoul, South Korea; ^9^Department of Neurology, Chuncheon Sacred Heart Hospital, Hallym University College of Medicine, Chuncheon, South Korea; ^10^Department of Neurology, Korea University College of Medicine, Seoul, South Korea; ^11^Department of Neurology, Chungnam National University College of Medicine, Daejeon, South Korea; ^12^Department of Neurology, Uijeongbu St. Mary's Hospital, The Catholic University of Korea College of Medicine, Uijeongbu, South Korea; ^13^Department of Neurology, Inje University College of Medicine, Seoul, South Korea; ^14^Department of Neurology, Eulji University, Uijeongbu, South Korea; ^15^Department of Neurology, School of Medicine, Kyungpook National University, Daegu, South Korea; ^16^Department of Neurology, Gyeongsang National University College of Medicine and Gyeonsang National University Hospital, Jinju, South Korea; ^17^Dr. Choi's Neurology Clinic, Jeonju, South Korea; ^18^Department of Neurology, Chung-Ang University Hospital, Seoul, South Korea; ^19^Department of Neurology, Dongtan Sacred Heart Hospital, Hallym University College of Medicine, Hwaseong, South Korea

**Keywords:** headache, primary headache disorder, cluster headache, delayed diagnosis, Korea

## Abstract

**Objective:**

Cluster headache (CH) is a rare, primary headache disorder, characterized of excruciating, strictly one-sided pain attacks and ipsilateral cranial autonomic symptoms. Given the debilitating nature of CH, delayed diagnosis can increase the disease burden. Thus, we aimed to investigate the diagnostic delay, its predictors, and clinical influence among patients with CH.

**Methods:**

Data from a prospective multicenter CH registry over a 4-year period were analyzed. CH was diagnosed according to the International Classification of Headache Disorders (ICHD)-3 criteria, and diagnostic delay of CH was assessed as the time interval between the year of the first onset and the year of CH diagnosis. Patients were classified into three groups according to the tertiles of diagnostic delay (1st tertile, <1 year; 2nd tertile, 1–6 years; and 3rd tertile, ≥7 years).

**Results:**

Overall, 445 patients were evaluated. The mean duration of diagnosis delay was 5.7 ± 6.7 years, (range, 0–36 years). Regarding the age of onset, majority of young patients (age <20 years) belonged to the third tertile (60%), whereas minority of old patients (>40 years) belonged to the third tertile (9.0%). For year of onset, the proportion of patients in the 3rd tertile was the highest for the groups before the publication year of the ICHD-2 (74.7%) and the lowest for the groups after the publication year of the ICHD-3 beta version (0.5%). Compared with the first CH, episodic CH [multivariable-adjusted odds ratio (aOR) = 5.91, 95% CI = 2.42–14.48], chronic CH (aOR = 8.87, 95% CI = 2.66–29.51), and probable CH (aOR = 4.12, 95% CI = 1.48–11.43) were associated with the tertiles of diagnostic delay. Age of onset (aOR = 0.97, 95% CI = 0.95–0.99) and PHQ-9 score (aOR = 0.96, 95% CI = 0.93–0.99) were inversely associated with the tertile of diagnostic delay. The prevalence of suicidal ideation was highest in the patients of the third tertile. The mean HIT-6 score increased significantly with the diagnostic delay (*p* = 0.041).

**Conclusions:**

Patients with a younger onset of CH have a higher risk of diagnostic delay. Nevertheless, the rate of delayed diagnosis gradually improved over time and with the publication of the ICHD criteria, supporting the clinical significance of diagnostic clinical criteria and headache education to reduce the disease burden of CH.

## Introduction

Cluster headache (CH) is rare but is the most painful primary headache disorder. It is characterized by recurrent excruciating pain attacks accompanied by ipsilateral cranial autonomic features (1, 2). The pain attacks of CH tend to occur on a circadian rhythm and maintain between 15 and 180 min from every other day to eight times a day within bouts and usually last several weeks. The main features of CH are distinct from those of migraine and other primary headache disorders. The current clinical criteria of CH in the International Classification of Headache Disorders (ICHD) sensibly reflect those distinguishing clinical features (3, 4).

Despite accumulating evidence on the pathophysiology of CH, there is no reliable biomarker available for its diagnosis (1, 2, 5, 6). Therefore, history-taking and clinical presentation are currently the only strategy for an accurate clinical diagnosis of CH. Furthermore, early diagnosis of CH remains a challenge because many patients with CH experience delayed or misdiagnosis (7, 8). Given the debilitating nature of CH, its diagnostic delay or misdiagnosis can seriously increase the disease burden and negatively affect quality of life. Nonetheless, predictors of diagnostic delay of CH and its clinical influence have not been extensively studied.

Therefore, the purpose of this study was to investigate the diagnostic delay of CH and its associated factors, using a relatively large-sized registry of patients with CH. We also evaluated the influence of delayed diagnosis on psychiatric comorbidities, suicidal ideation and attempt, and headache impact.

## Materials and Methods

### Study Design and Patients

The Korean Cluster Headache Registry (KCHR) study is a prospective, multicenter registry that includes patients with consecutive CH aged ≥19 years across Korea. The KCHR version 1 enrolled patients between September 2016 and December 2018, and the KCHR version 2 commenced enrollment on October 2018 from 15 university hospitals (nine tertiary and six secondary referral centers) and two secondary referral general hospitals following the Institutional Review Board (IRB) approval in each research hospital.

The current study is cross-sectional and planned as part of the KCHR study. Herein, the study population recruited between September 2016 and December 2020 was evaluated. The detailed protocol of the KCHR has been previously published (9–11). This study was reviewed and approved by the IRB at each study hospital and complied with the Declaration of Helsinki and Good Clinical Practice guidelines. All patients understood the study objectives and provided written informed consent before their voluntary participation.

All patients were carefully evaluated by KCHR investigators in person, who are experienced board-certified neurologists specialized in headache disorders. CH was diagnosed based on the patient's history and clinical presentation using the third edition, beta version of the ICHD (ICHD-3β) and the third edition of the ICHD (ICHD-3). Among the recruited patients, only those with CH compatible with the ICHD-3 criteria were included in the present study. CH subtype was classified according to the ICHD-3. Patients who did not experience remission within 1 year of their first CH episode or those who did not follow more than 1 year were classified to either have episodic CH (ECH) or chronic CH (CCH) and were further classified into first CH. For these patients, the diagnosis was finally coded as 3.1.

### Data Collection and Measurements

Data on demographics and social habits, headache diagnosis, CH history and characteristics, psychiatric status, suicidal ideation and attempt, and headache impact were collected. Diagnostic delay of CH was defined as the time interval between the year of the first onset and the year of CH diagnosis. The 12-item Allodynia Symptom Checklist was used to assess cutaneous allodynia during pain attacks of CH (12). Anxiety and depression were evaluated using the Korean versions of the Generalized Anxiety Disorder 7-item scale (GAD-7) and the Patient Health Questionnaire 9-item scale (PHQ-9). Generalized anxiety disorder (GAD) and major depressive disorder (MDD) were defined as a GAD-7 score of ≥10 and PHQ-9 score of ≥10, respectively (13). Suicidal ideation and attempt were assessed by two individual lucid questions (“Have you ever thought that it was better to die?” and “Have you ever attempted suicide?”, respectively). Headache impact was measured using the 6-item Headache Impact Test (HIT-6) (14).

### Statistical Analysis

For practical analysis, the patients with CH were classified into three groups according to the tertile of diagnostic delay (1st tertile, <1 year; 2nd tertile, 1–6 years; and 3rd tertile, ≥7 years). Continuous variables were presented as the mean ± SD, whereas categorical variables were presented as numbers (percentages). The statistical significance of intergroup differences were analyzed using the one-way ANOVA test for continuous variables and the chi-square test for categorical variables. Ordinal logistic regression analysis was performed to identify the factors associated with the tertile of diagnostic delay. The results of the univariate analyses were presented as odds ratio (OR) and 95% CI. Significant variables in the univariable analyses (*p* < 0.05) were entered into multivariable models to confirm their independent relationships. In terms of sample size, *post-hoc* power analysis based on comparison of prevalence of diagnostic delay ≥1 year between two groups (80.9% in the group of age of onset <30 years, no. = 262 vs. 53.6% in the group of age of onset ≥30 years, no. = 183) valued at 100. Independent influencing factors were presented with multivariable-adjusted odds ratio (aOR) and 95% CI. All statistical analyses were performed using SPSS 18.0 (SPSS Inc., Chicago, IL. USA). All reported *p*-values were two-tailed, and *p* < 0.05 was considered significant.

## Results

### Patient Characteristics and Diagnostic Delay of CH

**Figure 1:**
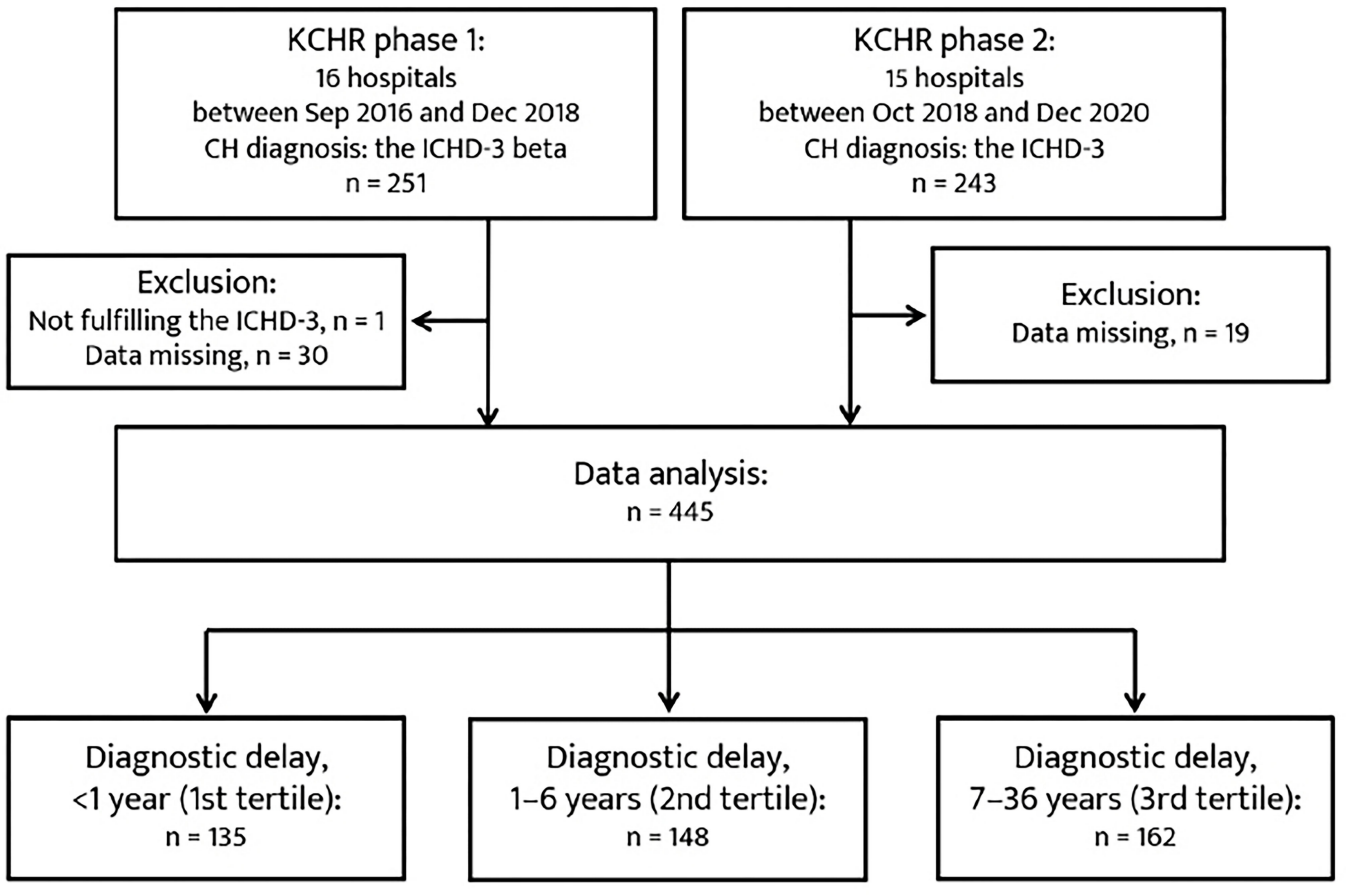
Patient recruitment flowchart. CH, cluster headache; ICHD-3, Third edition of the International Classification of Headache Disorders.

A total of 445 patents were included in the analysis (Figure 1). The mean duration of diagnosis delay was 5.7 ± 6.7 years (range, 0–36 years). In total, 135, 148, and 162 patients were classified into the 1st, 2nd, and 3rd tertiles of diagnostic delay, respectively. The baseline characteristics of the patients in the tertiles of diagnostic delay are summarized in Table 1. Regarding CH subtype, 150 patients with ECH (45.7%) were in the 3rd tertile group, and 12 patients with CCH (63.2%) were in the 2nd tertile group. The patients in the 3rd tertile group had a younger age of onset of CH, longer lifetime disease duration, and a higher number of lifetime cluster bouts. Furthermore, their year of onset of CH was earlier than the 1st and 2nd tertile groups.

**Table 1:** Baseline characteristics stratified by the tertile of diagnostic delay of cluster headache (CH) among patients with CH.

	**Diagnostic delay**			
	**1st tertile**,	**2nd tertile**,	**3rd tertile**,	***p***
	** <1 year**	**1–6 years**	**7–36 years**	
	**(*N* = 135)**	**(*N* = 148)**	**(*N* = 162)**	
**Demographics and social habits**				
Age, year	38.1 ± 11.5	35.2 ± 10.7	38.3 ± 9	0.017
Female sex, no. (%)	33 (41.3)	23 (28.8)	24 (30)	0.063
BMI, kg/m^2^	24.1 ± 3.4	24.2 ± 3	23.9 ± 3.2	0.781
Current smoking, no. (%)	62 (31.8)	62 (31.8)	71 (36.4)	0.792
Alcohol drinking, no. (%)	72 (28.8)	84 (33.6)	94 (37.6)	0.709
**Diagnosis**				
CH subtype, no. (%)				<0.001
Episodic CH	61 (18.6)	117 (35.7)	150 (45.7)	
Chronic CH	4 (21.1)	12 (63.2)	3 (5.8)	
Probable CH	22 (51.2)	13 (30.2)	8 (18.6)	
First CH	48 (87.3)	6 (10.9)	1 (1.8)	
Coexisting migraine, no. (%)	25 (37.3)	23 (34.3)	19 (28.4)	0.26
**Disease history**				
Age of onset, years	34.3 ± 11.6	29.1 ± 11.6	23.2 ± 8.3	<0.001
Year of onset	2,014 ± 5	2,012 ± 4	2,003 ± 6	<0.001
Lifetime disease duration, year	3.8 ± 5.7	6 ± 5.1	15 ± 6.3	<0.001
Lifetime cluster bout	4.3 ± 6.8	8.1 ± 13.2	12.3 ± 11.9	<0.001
**Disease characteristics**				
Attack severity (0–10 NRS)	8.8 ± 1	8.6 ± 1.5	9.1 ± 1	0.011
Attack frequency per day	2.4 ± 2.5	1.8 ± 1.5	1.9 ± 2	0.05
Attack duration, mins	98.1 ± 74.2	101.9 ± 69	116 ± 122.9	0.215
Diurnal rhythmicity, no. (%)	83 (31.2)	84 (31.6)	99 (37.2)	0.656
Seasonal rhythmicity, no. (%)	39 (19.7)	70 (35.4)	89 (44.9)	<0.001
ASC score	1.5 ± 3	1.1 ± 2.6	1.6 ± 3.1	0.261
**Psychiatric status and headache impact**				
GAD-7 score	8.5 ± 5.8	7.1 ± 5.8	8 ± 6	0.144
PHQ-9 score	9.4 ± 6.3	7.2 ± 6.3	7.6 ± 6.8	0.013
Suicidal ideation, no. (%)	26 (19.7)	21 (35.4)	46 (44.9)	0.008
Suicidal attempt, no. (%)	2 (50)	0 (0)	2 (50)	0.881
HIT-6 score	67.4 ± 9.3	68.1 ± 8.1	69.8 ± 7.6	0.039

*Plus–minus values present mean ± SD*.

*ASC, 12-item Allodynia Symptom Checklist; BMI, body mass index; CH, cluster headache; GAD-7 Generalized Anxiety Disorder (7-item scale); HIT-6, 6-item Headache Impact Test; NRS, numerical rating scale; PHQ-9 Patient Health Questionnaire (9-item scale)*.

**Figure 2:**
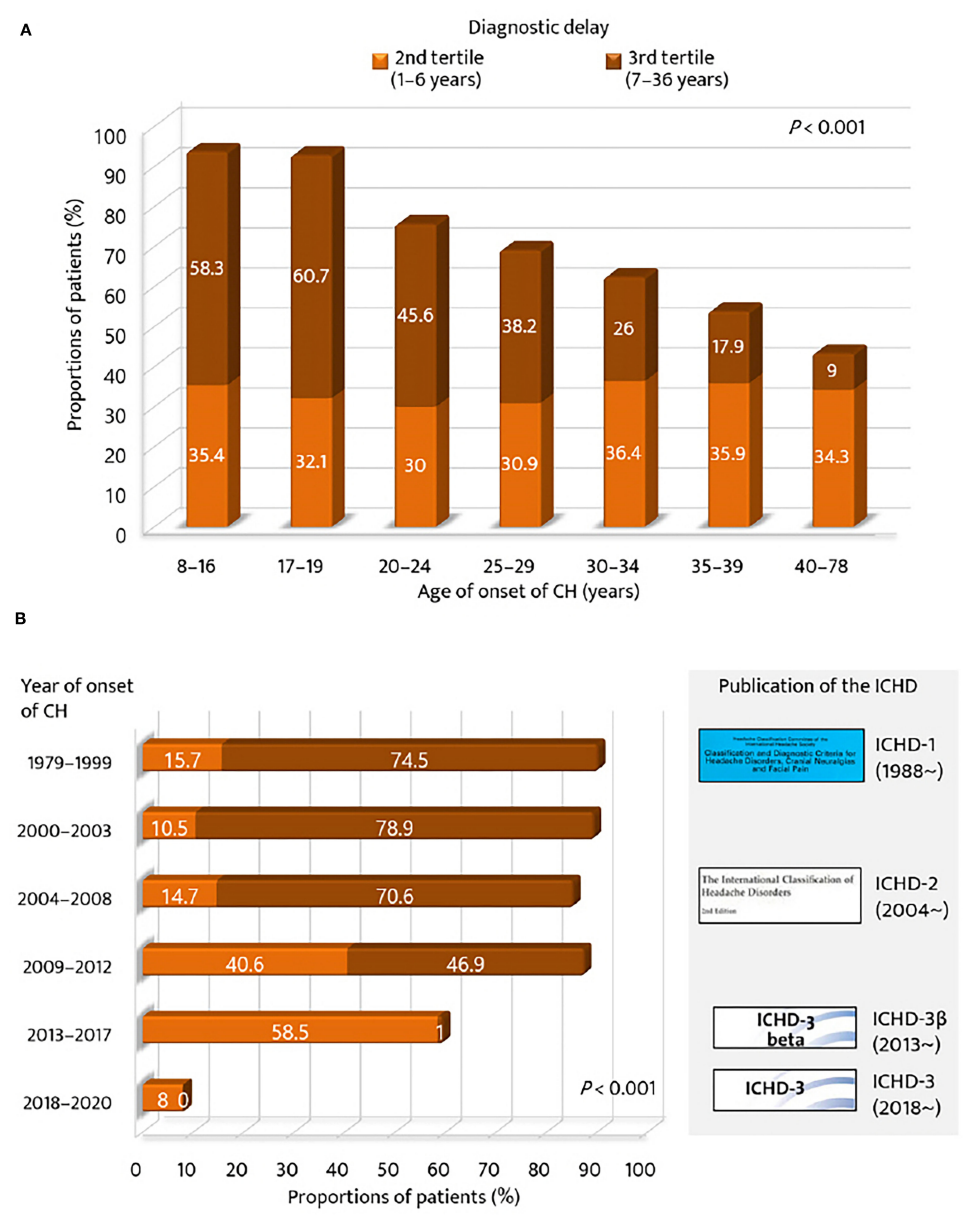
Proportions of patients with diagnostic delay of cluster headache ≥1 year. According to **(A)** age of onset of cluster headache and **(B)** year of onset of cluster headache. GAD-7, Generalized Anxiety Disorder (seven-item scale); ICHD, International Classification of Headache Disorders.

Regarding the age of onset of CH, the proportion of the 2nd and 3rd tertile groups of diagnostic delay was compared across the strata of age of CH onset (Figure 2A). The proportion of patients in the 3rd tertile was the highest for the younger age groups (<20 years, 60%), but the lowest for the older age group (>40 years, 9.0%). The proportion of patients with a diagnostic delay ≥1 year was gradually decreased with age (*p* < 0.001). For year of onset, the 2nd and 3rd tertile groups of diagnostic delay of CH were compared with respect to the strata of time and the publication of the ICHD criteria (Figure 2B). The proportion of patients in the 3rd tertile was the highest for the groups before the publication year of the ICHD-2 (74.7%) but the lowest for the groups after the publication year of the ICHD-3β version (0.5%). The proportion of patients with a diagnostic delay of CH ≥1 year significantly decreased with time and the publication of the ICHD (*p* < 0.001).

### Factors Associated With the Tertile of Diagnostic Delay

In univariable analyses of CH subtype, ECH, CCH, and probable CH were significantly associated with the tertile of diagnostic delay of CH, compared to the first CH. Among CH subtypes, ECH had the highest risk of diagnostic delay, with an OR two times higher than that of CCH (OR = 30.05, 95% CI = 13.68–65.95; Table 2). Coexisting migraine had a marginal association with the tertile of diagnostic delay (OR = 0.63, 95% CI = 0.40–1.00; *p* = 0.053), and this did not reach statistical significance. Lifetime disease duration, lifetime cluster bout, seasonal rhythmicity, suicidal ideation, and HIT-6 score were inversely associated with the tertile of diagnostic delay. Female sex, age of onset, year of onset, and PHQ-9 score were also inversely associated with the tertile of diagnostic delay.

**Table 2:** Ordinal logistic regression analysis: independent variables of the tertile of diagnostic delay of cluster headache.

	**Unadjusted**		**Model 1**		**Model 2**	
	**OR (95% CI)**	***p***	**aOR (95% CI)**	***p***	**aOR (95% CI)**	***p***
Age, year	1.00 (0.98–1.01)	0.854				
Female sex	0.63 (0.41–0.97)	0.038	0.74 (0.45–1.23)	0.254	0.76 (0.44–1.29)	0.314
BMI, kg/m^2^	0.99 (0.94–1.05)	0.968				
Current smoking	0.89 (0.63–1.24)	0.501				
Alcohol drinking	1.23 (0.88–1.72)	0.212				
**CH subtype**						
Episodic CH	30.05 (13.68–65.95)	<0.001	6.00 (2.55–14.06)	<0.001	5.91 (2.42–14.48)	<0.001
Chronic CH	14.18 (4.62–43.42)	<0.001	8.53 (2.64–27.57)	<0.001	8.87 (2.66–29.51)	<0.001
Probable CH	6.38 (2.51–16.21)	<0.001	3.65 (1.38–9.69)	0.009	4.12 (1.48–11.43)	0.006
First CH	reference		reference		reference	
Coexisting migraine	0.63 (0.40–1.00)	0.053				
Age of onset, year	0.92 (0.91–0.94)	<0.001	0.97 (0.95–0.99)	0.01	0.97 (0.95–0.99)	0.033
Year of onset	0.78 (0.75–0.81)	<0.001	0.96 (0.81–1.14)	0.688	0.97 (0.80–1.17)	0.775
Lifetime disease duration, year	1.27 (1.22–1.32)	<0.001	1.16 (0.97–1.38)	0.091	1.17 (0.97–1.42)	0.093
Lifetime cluster bout	1.08 (1.05–1.11)	<0.001	1.00 (0.98–1.01)	0.969	1.00 (0.98–1.01)	0.981
Attack severity (0–10 NRS)	1.13 (0.99–1.29)	0.062				
Attack frequency per day	0.92 (0.85–1.00)	0.078				
Attack duration, mins	1.01 (1.00–1.01)	0.107				
Diurnal rhythmicity	1.01 (0.72–1.41)	0.943				
Seasonal rhythmicity	2.27 (1.62–3.19)	<0.001	1.07 (0.71–1.60)	0.731	1.00 (0.67–1.51)	0.962
ASC score	1.00 (0.95–1.06)	0.765				
GAD-7 score	0.99 (0.96–1.02)	0.668				
PHQ-9 score	0.97 (0.94–0.99)	0.028			0.96 (0.93–0.99)	0.033
Suicidal ideation	1.58 (1.04–2.41)	0.03			1.03 (0.60–1.76)	0.898
HIT-6 score	1.02 (1.01–1.05)	0.005			1.02 (0.99–1.05)	0.089

*Significant variables (p < 0.05 at univariable analyses) were selected to develop multivariable models*.

*Model 1 includes significant variables of demographics, social habits, diagnosis, disease history, and disease characteristics*.

*Model 2 was additionally adjusted for significant variables of psychiatric status and headache impact*.

*aOR, multivariable-adjusted odds ratio; ASC, 12-item Allodynia Symptom Checklist; BMI, body mass index; CH, cluster headache; GAD-7 Generalized Anxiety Disorder (7-item scale); HIT-6, 6-item Headache Impact Test; NRS, numerical rating scale; PHQ-9, Patient Health Questionnaire (9-item scale)*.

A multivariable-adjusted model was then constructed by entering the following significant variables in the univariable analyses: female sex, CH subtype, age of onset, year of onset, lifetime disease duration, lifetime cluster bout, seasonal rhythmicity, PHQ-9 score, suicidal ideation, and HIT-6 score. In contrast to the results of univariable analyses, female sex, year of onset, lifetime disease duration, lifetime cluster bout, seasonal rhythmicity, suicidal ideation, and HIT-6 score were not significantly associated with the diagnostic delay of CH in the multivariable-adjusted models. In model 2, fully adjusting for all the significant variables in the univariable analyses, CH subtype (aOR = 5.91, 95% CI = 2.42–14.48 for ECH; aOR = 8.87, 95% CI = 2.66–29.51 for CCH; and aOR = 4.12, 95% CI = 1.48–11.43 for probable CH), age of onset (aOR = 0.97, 95% CI = 0.95–0.99), and PHQ-9 score (aOR = 0.96, 95% CI = 0.93–0.99) remained as significant predictors of diagnostic delay. Regarding CH subtype, the risk of diagnostic delay of CH for CCH was 1.5 times higher than that for ECH, in contrast to the result of univariable analysis.

### Association of Diagnostic Delay With Anxiety, Depression, Suicidal Ideation, and Headache Impact

**Figure 3:**
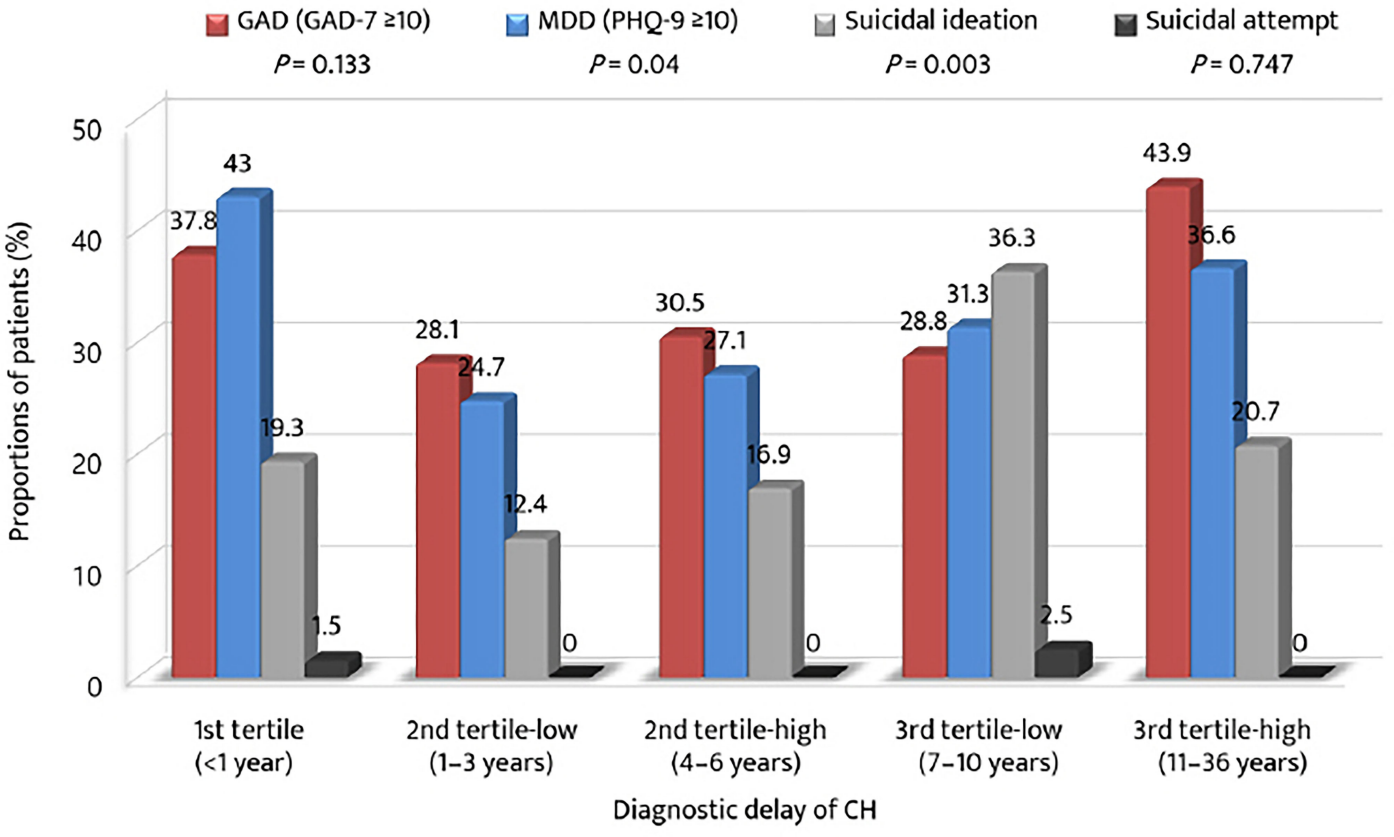
Proportions of anxiety, depression, suicidal ideation, and suicidal attempt among the tertiles of diagnostic delay of cluster headache. GAD-7, Generalized Anxiety Disorder (seven-item scale); PHQ-9, Patient Health Questionnaire (9-item scale); MDD, major depressive disorder.

The proportions of patients with anxiety, depression, and suicidal ideation were compared in accordance with the strata of the tertile of diagnostic delay (Figure 3). The prevalence of GAD was the highest in the 3rd tertile-high group, whereas the prevalence of MDD was the highest in the 1st tertile group. The prevalence of suicidal ideation was the highest in the 3rd tertile-low group. Except for the 1st tertile group, the proportions of GAD, MDD, and suicidal ideation increased from the 2nd tertile-low to the 3rd tertile-low or high groups.

**Figure 4:**
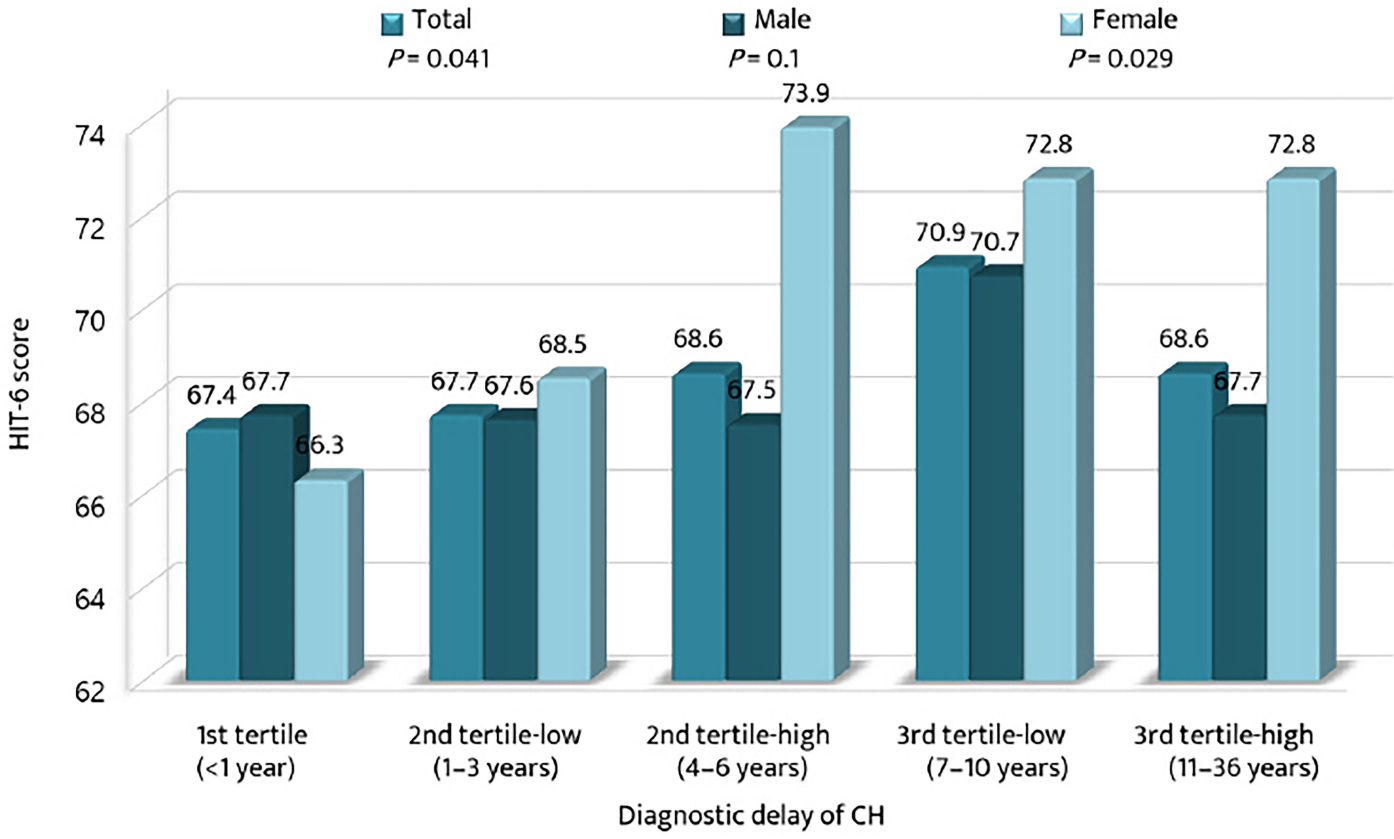
Comparison of the mean scores of the 6-item Headache Impact Test among the tertiles of diagnostic delay of cluster headache. CH, cluster headache; HIT-6, the 6-item Headache Impact Test.

The mean HIT-6 scores among the tertile groups of diagnostic delay were compared with respect to sex (Figure 4). The mean HIT-6 score increased significantly with the diagnostic delay of CH (*p* = 0.041). The HIT-6 score was the highest in the 3rd tertile-low group for men and in the 2nd tertile-high group for women. For the 2nd tertile-low and the 3rd tertile-high groups, the mean HIT-6 scores were higher for women than for men.

## Discussion

In the present study, 36.4% of patients experienced a diagnostic delay of CH for ≥7 years. The diagnostic delay decreased in the recent decade, especially after the publication of the ICHD-3β criteria. In the multivariable analyses, age of onset, PHQ-9 score, and CH subtype were independent predictors of diagnostic delay of CH. With respect to psychological profiles, the proportion of patients with suicidal ideation was higher in patients with prolonged diagnostic delay than those with moderate diagnostic delay. In addition, patients with CH with longer diagnostic delay had a higher headache impact, especially for female patients.

The mean diagnostic delay of CH was 5.7 years, which is within the range of previous clinical studies in East and West (2.6–9 years), but shorter than that from the studies in Far Eastern countries (Japan, Taiwan, and China; 7.3–9.3 years) (7, 15–18). Given the similarities of ethnicity, culture, and healthcare insurance between the countries, one plausible explanation for the shorter diagnostic delay in our cohort is that the most of the previous studies were conducted before the publication of the ICHD-3β version. In our cohort, the diagnosis was delayed for a mean of 9.14 years in patients whose disease onset was before 2013, and this is comparable with the range from previous Far Eastern studies (15, 17, 18). There is currently no confirmatory modality for CH; thus, clinical criteria proposed in the ICHD are used. A newer version of the ICHD criteria may promote educational activities and enhance interest in major headache disorders and improve diagnostic delay of CH (19). A decrease in diagnostic delay as the year of CH onset advanced has been reported in previous studies (7, 16). In this regard, our findings may highlight the clinical significance of clinical criteria and disease awareness for reducing the diagnostic delay of CH (20, 21).

The diagnostic delay of CH is attributed to patients and their headache characteristics. Borderline characteristics between migraine and CH may increase confusion regarding headache diagnosis. Accordingly, some studies have investigated these characteristics to identify the predictors of diagnostic delay of CH (22–24). The studies compared the clinical details between patients with and without diagnostic delay and reported that younger onset, earlier year of onset, ECH, delayed maximum pain intensity exceeding the first 5 min, migraine features, nocturnal attacks, restlessness, radiating jaw pain, alternation of attack side, side shifting of pain between bouts, and absence of cranial autonomic symptoms delayed the correct diagnosis. In a Danish study, univariable analyses demonstrated that diagnostic delay was associated with the year of onset after 1990, prolonged attack duration >180 min, migraine-like features, and nocturnal attacks (16).

With respect to the CH subtype, it has been traditionally believed that diagnostic delay is associated with ECH with a short bout period and extended remission period, compared to CCH (14). Previous studies did not confirm this concept. In our study, the risk of diagnostic delay of ECH was two times higher than that of CCH in the univariate analysis, but this was reversed after the multivariable adjustment. These results suggest that the effect of CH subtype (i.e., ECH vs. CCH) on diagnostic delay may be similar. Nonetheless, for the Asian population, CCH may be harder to diagnose early because of its rarity and poor recognition of this subtype (15, 17, 18, 25).

In line with previous studies, we found that early age of onset is a risk factor for diagnostic delay. Particularly, more than 90% of the patients with adolescent onset (≤ 19 years) experienced a diagnostic delay of ≥1 year. The exact reason is still unknown, but previous studies have assumed that misunderstanding of the onset age of CH, especially adolescent onset of CH, and higher suspicion of serious secondary headache disorders among patients with late onset (age ≥40 years at onset) affect the pattern of diagnostic delay according to age of CH onset (7, 16, 22, 26). Further studies are needed to clarify this. Finally, higher PHQ-9 scores were associated with an earlier diagnosis of CH. One possible reason for this association is that depression can amplify pain perception, and CH patients with depression may seek a correct diagnosis and medical management more aggressively (27). In this regard, the inverse association between the PHQ-9 score and diagnostic delay suggests that comorbid depression in the early period of CH may facilitate earlier diagnosis of CH. This assumption should be confirmed in additional studies.

With regard to psychiatric comorbidities, the proportions of patients with anxiety, depression, and suicidal ideation were higher in the 1st and 3rd tertiles. Except for patients with an earlier diagnosis (<1 year), the prevalence of anxiety, depression, and suicidal ideation gradually increased across the tertiles of diagnostic delay. These findings suggest that an extended period of untreated CH may have a negative impact on affective aspects. Recent studies have shown CH is basically a risk factor of psychiatric comorbidities (28–30). This could be due to the shared neurobiology and anatomical location of the pain matrix and depression processing site. Consequently, a longer diagnostic delay may be worsening condition, in terms of psychiatric aspects. In this context, clinicians need to pay more attention to the psychiatric comorbidities of patients with CH with delayed diagnosis. Regarding headache impact, the impact was higher in groups with diagnostic delay. This finding highlights the importance of reducing diagnostic delay in CH. Notably, headache impact was greater in female patients with prolonged diagnostic delays. Hence, we need to remember that although there were fewer female patients with CH than male patients, reducing their diagnostic delay should be a priority.

This study has some limitations that need to be considered. First, our study was based on a hospital-based cohort registry. This raises the possibility of underestimation of the number of patients with CH with delayed diagnosis because some patients with CH do not visit the hospital or are incorrectly diagnosed or managed. Therefore, future population-based studies are warranted to further evaluate the exact prevalence and impact of delayed diagnosis of CH. Second, our study did not analyze the misdiagnosis or mismanagement of patients with delayed diagnosis. However, these are important issues in CH because misdiagnosis or mismanagement may be direct causes of delayed diagnosis of CH (7). Further comprehensive studies on delayed diagnosis, misdiagnosis, and mismanagement of CH are required. Third, multivariate logistic regression analyses was not adjusted for unmeasured potential confounders, such as socioeconomic status, educational level, sleep disorders, and history of previous misdiagnosis or mismanagement of CH. These should be considered in future multivariable analyses. Fourth, some measurement (i.e., age of onset, onset of year, lifetime disease duration, and number of lifetime cluster bout) was based on a retrospective data collection. Therefore, the possibility of recall bias should be considered in the interpretation of the results. Finally, suicidal idea was not assessed by the well-validated tool like the Columbia-Suicide Severity Rating Scale. Further studies need to use such tool for the analysis of suicidal ideation (31).

In conclusion, two-thirds of the patients with CH were diagnosed at least 1 year after the onset of CH. This indicates that diagnostic delay remains a hallmark of CH. The rate of diagnostic delay of CH gradually decreases over time and with the publication of the ICHD criteria. This supports the clinical significance of diagnostic criteria and headache education in reducing the delayed diagnosis of CH. A younger age at onset is a risk factor for delayed diagnosis, suggesting the need to pay close attention to the headache diagnosis of patients with CH with younger age at onset. The negative influence of diagnostic delay on psychiatric comorbidities and headache impact should also be considered in patients with CH.

## Data Availability Statement

The original contributions presented in the study are included in the article/supplementary materials, further inquiries can be directed to the corresponding author.

## Ethics Statement

The studies involving human participants were reviewed and approved by Bundang Jesaeng General Hospital, Kangbuk Samsung Hospital, Eulji Hospital, Samsung Medical Center, Severance Hospital, Seoul Medical Center, The Catholic University of Korea, Seoul Hospital, Ewha Womans University Seoul Hospital, Chuncheon Sacred Heart Hospital, Korea University College of Medicine, Chungnam National University College of Medicine, Uijeongbu St. Mary's Hospital, Inje University College of Medicine, Eulji University, Uijeongbu, Kyungpook National University, Gyeongsang National University College of Medicine and Gyeonsang National University Hospital, Chung-Ang University Hospital, Dongtan Sacred Heart Hospital. The patients/participants provided their written informed consent to participate in this study.

## Author Contributions

BSK and SJC devised the idea and designed the study for this article and drafted the initial manuscript. BSK, PWC, BKK, ML, MC, JYA, DB, TJS, JHS, KO, DK, JMK, JP, JC, HSM, SC, JGS, SKK, YJC, KYP, CSC, and SJC contributed the acquisition of the data and conducted the data analyses and data interpretations. PWC, BKK, ML, MC, JYA, DB, TJS, JHS, KO, DK, JMK, JP, JC, HSM, SC, JGS, SKK, YJC, KYP, and CSC made the critical revision of the paper with important intellectual content. All authors reviewed and approved the final version of the manuscript and agreed to be responsible aspects of the work in ensuring that questions related to the accuracy or integrity of any part of the work are appropriately investigated and resolved.

## Conflict of Interest

SJC was involved as a site investigator of multicenter trial sponsored Otsuka Korea, Allergan, Ildong Pharmaceutical Co., Ltd., Novartis International AG, Eli Lilly and Company, Hyundaipharm. Co. Ltd., Biohaven Asia Pacific Ltd., H. Lundbeck A/S (Lundbeck), and Parexel Korea Co., Ltd., and received lecture honoraria from Allergan Korea, WhanIn Pharm Co., Ltd., Shinpoong Pharma. Co., Ltd., and SK chemicals in the past 24 months. MC was a site investigator for a multicenter trial sponsored by Otsuka Korea, Novartis International AG, and Eli Lilly and Company. He worked as an advisory member for Teva and has received lecture honoraria from Allergan Korea, Handok-Teva, and Yuyu Pharmaceutical Company in the past 24 months. He received grants from the Yonsei University College of Medicine and the National Research Foundation of Korea (2019R1F1A1053841). The remaining authors declare that the research was conducted in the absence of any commercial or financial relationships that could be construed as a potential conflict of interest.

## Publisher's Note

All claims expressed in this article are solely those of the authors and do not necessarily represent those of their affiliated organizations, or those of the publisher, the editors and the reviewers. Any product that may be evaluated in this article, or claim that may be made by its manufacturer, is not guaranteed or endorsed by the publisher.

## Abbreviations

aOR: multivariable-adjusted odds ratio
CCH: chronic cluster headache
CH: cluster headache
CI: confidence interval
ECH: episodic cluster headache
GAD: generalized anxiety disorder
GAD-7: the Generalized Anxiety Disorder 7-item scale
HIT-6: the 6-item Headache Impact Test
ICHD: the International Classification of Headache Disorders
ICHD-3: the third edition of the International Classification of Headache Disorders
IRB: institutional review board
KCHR: Korean cluster headache registry
MDD: major depressive disorder
OR: odds ratio
PHQ-9: the Patient Health Questionnaire 9-item scale.
